# P-915. Categorization and Temporal Dynamics of Mortality in All-Cause Encephalitis

**DOI:** 10.1093/ofid/ofae631.1106

**Published:** 2025-01-29

**Authors:** Jennifer Makhoul, Ashley N Heck, Paris Bean, Rajesh Gupta, Ralph Habis, John Probasco, Arun Venkatesan, Rodrigo Hasbun

**Affiliations:** University of Texas Health Science Center at Houston, Houston, Texas; McGovern Medical School, UTHealth Science Center, Houston, TX, Houston, Texas; UTHealth Science Center at Houston, Houston, Texas; UTHealth Science Center at Houston, Houston, Texas; Johns Hopkins University School of Medicine, Baltimore, Maryland; Johns Hopkins University School of Medicine, Baltimore, Maryland; Johns Hopkins University School of Medicine, Baltimore, Maryland; UT Health Mc Govern Medical School, Houston, Texas

## Abstract

**Background:**

Encephalitis in adults is associated with inpatient mortality in approximately 10% of patients but the cause and timing of death are unknown.

Variables associated with inpatient mortality in 641 adults with encephalitis.

**Figure:**
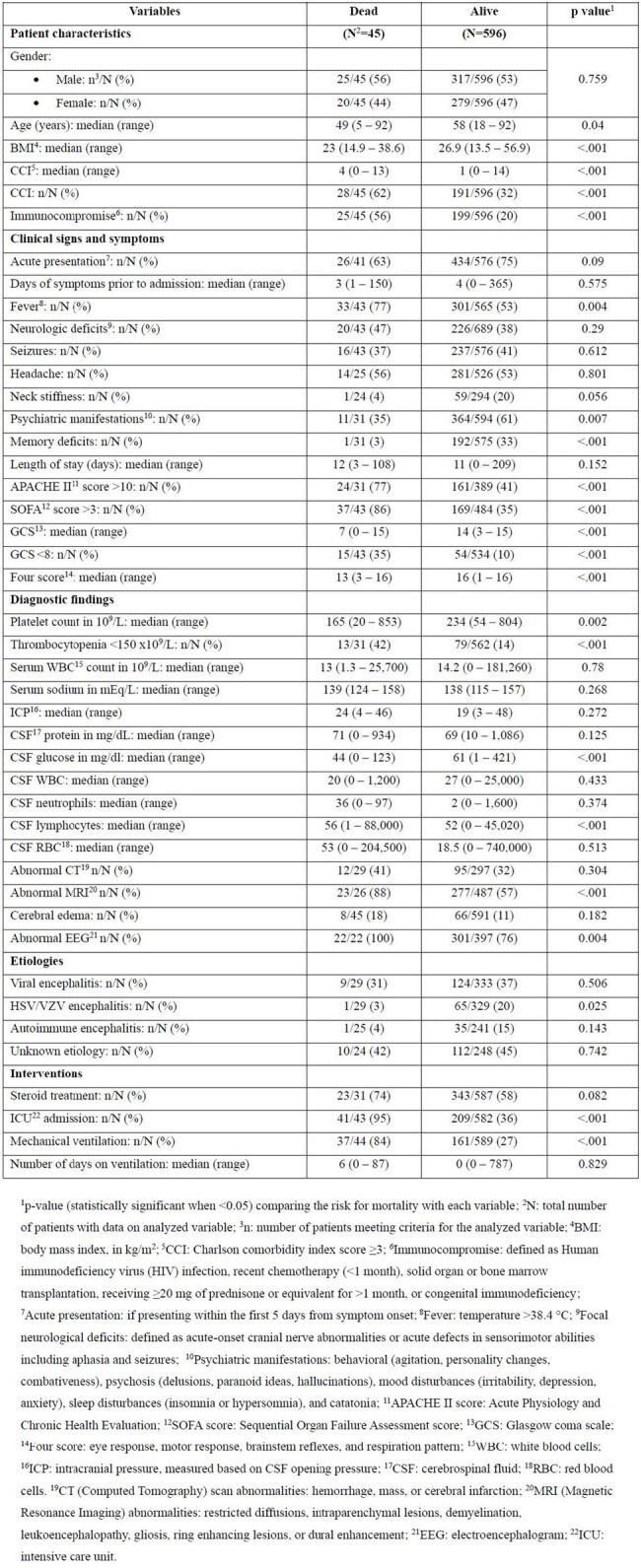

1p-value (statistically significant when <0.05) comparing the risk for mortality with each variable; 2N: total number of patients with data on analyzed variable; 3n: number of patients meeting criteria for the analyzed variable; 4BMI: body mass index, in kg/m2; 5CCI: Charlson comorbidity index score ≥3; 6Immunocompromise: defined as Human immunodeficiency virus (HIV) infection, recent chemotherapy (<1 month), solid organ or bone marrow transplantation, receiving ≥20 mg of prednisone or equivalent for >1 month, or congenital immunodeficiency; 7Acute presentation: if presenting within the first 5 days from symptom onset; 8Fever: temperature >38.4 °C; 9Focal neurological deficits: defined as acute-onset cranial nerve abnormalities or acute defects in sensorimotor abilities including aphasia and seizures; 10Psychiatric manifestations: behavioral (agitation, personality changes, combativeness), psychosis (delusions, paranoid ideas, hallucinations), mood disturbances (irritability, depression, anxiety), sleep disturbances (insomnia or hypersomnia), and catatonia; 11APACHE II score: Acute Physiology and Chronic Health Evaluation; 12SOFA score: Sequential Organ Failure Assessment score; 13GCS: Glasgow coma scale; 14Four score: eye response, motor response, brainstem reflexes, and respiration pattern; 15WBC: white blood cells; 16ICP: intracranial pressure, measured based on CSF opening pressure; 17CSF: cerebrospinal fluid; 18RBC: red blood cells. 19CT (Computed Tomography) scan abnormalities: hemorrhage, mass, or cerebral infarction; 20MRI (Magnetic Resonance Imaging) abnormalities: restricted diffusions, intraparenchymal lesions, demyelination, leukoencephalopathy, gliosis, ring enhancing lesions, or dural enhancement; 21EEG: electroencephalogram; 22ICU: intensive care unit.

**Methods:**

We conducted a retrospective study across two tertiary healthcare systems in Texas and Maryland between 2002-2023. The causes of death were categorized according to the World Health Organization and National Center for Health Statistics into 3 groups: category 1, where encephalitis was the immediate cause of death; category 2, the underlying cause contributing to mortality but not directly responsible; or category 3, unrelated to the cause of death, acting independently. We also examined the timing of death from initial presentation, and the association of different variables with mortality.

Distribution of causes of death per category in 45 adults with encephalitis

**Figure:**
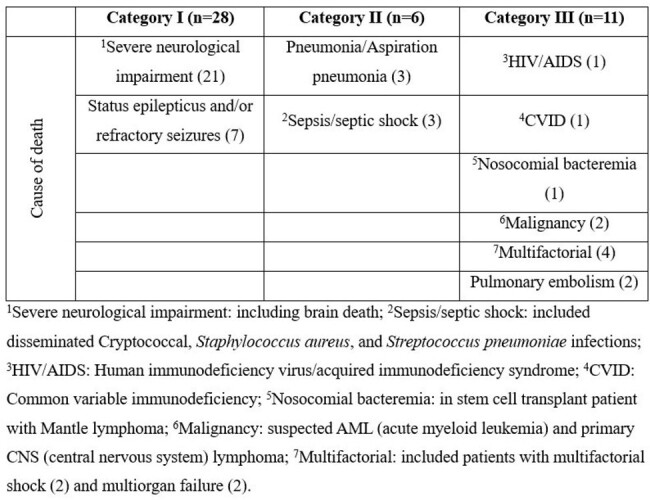

1Severe neurological impairment: including brain death; 2Sepsis/septic shock: included disseminated Cryptococcal, Staphylococcus aureus, and Streptococcus pneumoniae infections; 3HIV/AIDS: Human immunodeficiency virus/acquired immunodeficiency syndrome; 4CVID: Common variable immunodeficiency; 5Nosocomial bacteremia: in stem cell transplant patient with Mantle lymphoma; 6Malignancy: suspected AML (acute myeloid leukemia) and primary CNS (central nervous system) lymphoma; 7Multifactorial: included patients with multifactorial shock (2) and multiorgan failure (2).

**Results:**

A total of 641 adults who had encephalitis were enrolled, 45 (7%) had inpatient mortality. Baseline factors associated with mortality included age, low BMI, presence of comorbidities, immunocompromised state, fever, psychiatric manifestations, memory deficits, mechanical ventilation, altered mental status, thrombocytopenia, hypoglycorrhachia, lymphocytic pleocytosis, HSV or VZV etiology of encephalitis, abnormal electroencephalogram and abnormal magnetic resonance imaging of the brain (p < 0.05). Encephalitis was the immediate cause of death (category I) in 62% of cases with a median time to death of 12 days, the underlying (category 2) in 13% with a median time to death of 12.5 days, and unrelated (category 3) in 25% with a median time to death of 27 days.

Distribution of patients per category of death

**Figure:**
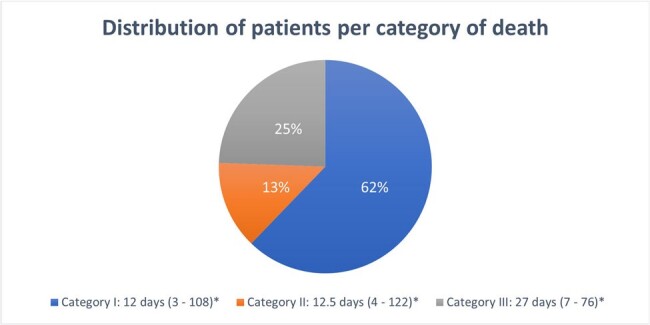

*Median timing of death, in days (range)

**Conclusion:**

Encephalitis was the immediate or underlying cause of inpatient mortality in the majority of patients, particularly in the first 14 days.

Evaluation of categories of death with timing of mortality

**Figure:**
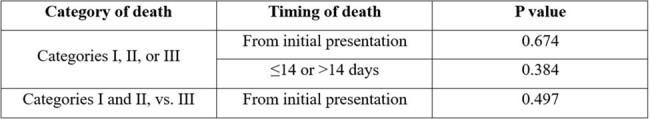

**Disclosures:**

**John Probasco, MD**, Genentech: Study site investigator for multicenter study **Rodrigo Hasbun, MD MPH FIDSA**, Biomeriaux: Grant/Research Support|Biomeriaux: Honoraria

